# Halo—A Universal Fluorescence Reader Based Threat Agent Detection Platform—A Proof of Concept Study Using SARS-CoV-2 Assays

**DOI:** 10.3389/fpubh.2022.852083

**Published:** 2022-04-12

**Authors:** Joseph Walish, Jason Cox, Jeremy Boone, Jennifer Stone, Nathan Henderson, Molly Maloney, Joe Ma, Jonathan Maa, Nghiem On, Konrad Petre, Bruce G. Goodwin, Shanmuga Sozhamannan, Robert Deans

**Affiliations:** ^1^C2Sense, Inc., Watertown, MA, United States; ^2^MRIGlobal, Kansas City, MO, United States; ^3^Maxim Biomedical, Inc., Rockville, MD, United States; ^4^Joint Program Executive Office for Chemical, Biological, Radiological and Nuclear Defense (JPEO-CBRND), Joint Project Lead for CBRND Enabling Biotechnologies (JPL CBRND EB), Frederick, MD, United States; ^5^Logistics Management Institute, Tysons, VA, United States

**Keywords:** SARS-CoV-2, diagnostics, rapid antigen test, immunoassay, fluorescence, europium, handheld reader, mobile app

## Abstract

Polymerase chain reaction (PCR) remains the gold standard in disease diagnostics due to its extreme sensitivity and specificity. However, PCR tests are expensive and complex, require skilled personnel and specialized equipment to conduct the tests, and have long turnaround times. On the other hand, lateral flow immunoassay-based antigen tests are rapid, relatively inexpensive, and can be performed by untrained personnel at the point of care or even in the home. However, rapid antigen tests are less sensitive than PCR since they lack the inherent target amplification of PCR. It has been argued that rapid antigen tests are better indicators of infection in public health decision-making processes to test, trace, and isolate infected people to curtail further transmission. Hence, there is a critical need to increase the sensitivity of rapid antigen tests and create innovative solutions to achieve that goal. Herein, we report the development of a low-cost diagnostic platform, enabling rapid detection of SARS-CoV-2 under field or at-home conditions. This platform (Halo™) is a small, highly accurate, consumer-friendly diagnostic reader paired with fluorescently labeled lateral flow assays and custom software for collection and reporting of results. The focus of this study is to compare the analytical performance of Halo^TM^ against comparable tests that use either colloidal gold nanoparticles or fluorescence-based reporters in simulated nasal matrix and not in clinical samples. Live virus data has demonstrated limit of detection performance of 1.9 TCID_50_/test in simulated nasal matrix for the delta variant, suggesting that single-assay detection of asymptomatic SARS-CoV-2 infections may be feasible. Performance of the system against all tested SARS CoV-2 virus variants showed comparable sensitivities indicating mutations in SARS-CoV-2 variants do not negatively impact the assay.

## Introduction

On March 11, 2020, the World Health Organization declared novel coronavirus COVID-19 a global pandemic^1^. Plague and pandemic in recent years are not unprecedented—diseases are traveling farther and faster thanks to a dramatically transient global population and highly interconnected global economies. Indeed, in just the last 20 years H1N1 (2009, death toll = 200,000), Ebola (2014, death toll = 11,000), SARS (2002, death toll = 770), and MERS (2015, death toll = 850) have been major epidemics threatening to become pandemics or full-fledged pandemics (1–3). Some are still ongoing; the HIV/AIDs pandemic has claimed the lives of >25 million people since 1981. What sets the COVID-19 pandemic apart from these other modern diseases is the rapid spread, relatively high number of deaths globally even with low mortality rates, and prevalence of asymptomatic carriers (4)^2^.

As of March 9th, 2022, more than 448 million cases and 6 million deaths have been reported globally from the COVID-19 pandemic^3^. With the rollout of multiple vaccines from Pfizer, Moderna, Astra Zeneca, and other government sponsored vaccines such as Sputnik, the world now has weapons that offer some protection against the serious side effects of the disease. Unfortunately, due to sustained and unhindered transmission of the virus, the risk of new variants and/or strains remain, and it is widely believed that COVID-19 is here to stay (5). An additional aid in the fight is the availability of widespread testing. Polymerase chain reaction (PCR) testing remains the gold standard in testing for the presence of the virus in clinical samples but is hindered by expensive and specialized equipment and the personnel needed to run the tests. Additionally, time delays in getting samples to the testing facility, analyzing them, and returning results, makes PCR tests less than ideal for use in public health decisions to curtail the spread. Rapid antigen tests (6–8) with results obtained in <20 mins are available and can be convenient but may not be sensitive enough to detect early stages of the disease when viral loads are low. Given this gap in sensitivity between the PCR and rapid tests and the need to obtain results as early as possible, there is a need for a technology to bridge the gap. The Halo SARS-CoV-2 Test aims to address this gap by offering a highly sensitive antigen test capable of confirming a COVID-19 infection within 20 mins through the use of a fluorescent immunoassay coupled with a low-cost reader using commercially available components to minimize cost and reduce the risk of supply chain issues upon scale-up.

Lateral flow assays (LFAs) are commonly used as rapid diagnostic tests and are well known as at-home pregnancy tests. Compared to traditional colorimetric (visually read) LFAs, fluorescent assays can greatly enhance the sensitivity of an assay when coupled with an appropriately designed reader. Conversion of an LFA test from colorimetric to fluorescent reporters has been shown to yield significant gains in performance, typically producing greater than 800% improvements in sensitivity (9–11). Because luminescence-based tests require a light source for excitation of the emitters, a reader is typically needed to supply this illumination as well as record the intensity of the light emitted from the fluorescent particles captured by the test and control lines. In addition to offering greater sensitivity, the use of a reader provides an opportunity to seamlessly compile results into an electronic database for reporting and surveillance purposes, critical for understanding the trajectory of a disease and influencing pandemic response decisions.

Here we describe a reader (Halo) that was developed to analyze and adjudicate the results of a lateral flow immunoassay that uses europium-based fluorescence reporters and a companion mobile phone app that displays the results and relays them to a cloud database. As a proof of concept, we report the results of a florescence based COVID-19 antigen LFA test using europium-labeled reporter beads and compare the results to an analogous gold nanoparticle-based visual test. We also present data on testing live SARS-CoV-2 wild type and variants viral stock and the impact of mutations present in the variants on assay performance.

### Halo Reader Design

**Figure 1:**
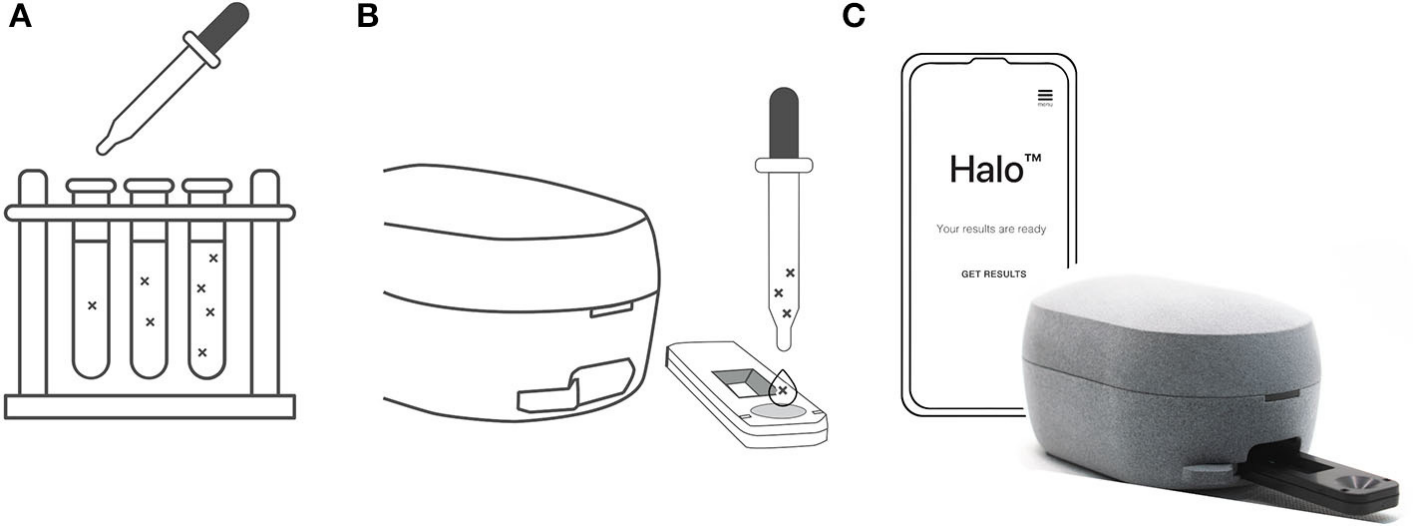
**(A)** SARS-CoV-2 stocks were diluted with simulated nasal matrix. Serial dilutions were then prepared to assess the preliminary limit of detection for each strain of the virus. **(B)** 80 μl of a given dilution was then added dropwise to the assay cassette via pipette and the assay developed for 15 mins. **(C)** The cassette was then inserted into the Halo reader to record the test line intensity.

The Halo reader uses a commercially available single-board computer (Raspberry Pi Zero 2 W) with Bluetooth capability coupled with a simple excitation source composed of a UV LED and driver circuit as well as an off-the-shelf CMOS (complementary metal-oxide-semiconductor) camera, used to acquire images which are then processed by the system. Any smartphone with Bluetooth connectivity can be used to interact with the reader through the use of an app. The result is a small, highly accurate, diagnostic reader that can be paired with fluorescently labeled lateral flow assays, and supportive software for consumer and/or professional use (Figure 1). By leveraging commercial-off-the-shelf (COTS) components and a simple circuit design, the cost of goods sold (COGS) for the reader is < $100 USD including assembly in the United States, when manufactured at volume.

The Halo Reader is a digitally connected solution with best-in-class sensitivity at an extremely reasonable cost. A key feature is the ability to use low-cost, readily available CMOS imagers to perform laboratory quality fluorescence measurements in both steady-state and time-delayed modes. This capability is fully compatible with existing commercially available emissive labels (e.g., europium beads) and therefore has access to the pipeline of LFAs targeting current and future challenges.

## Methods

### Sample Preparation, Testing, and General Usage of the Halo Reader

After unpacking the swab, reagent vial, and test cassette the user opens the Halo phone app and ensures that the appropriate lot-specific data for the assays has been obtained (either pre-loaded or downloaded from the cloud). Specimen collection is done through a nasal swab (anterior nares) or by laboratory prepared samples, as in this study (see Live Virus Testing). The specimen is then added to lysis buffer and mixed with the sample for 1 min to ensure any nucleocapsid protein present in the sample is exposed and able to be captured by the assay. Next, after the cassette is placed on a level surface, 80 μl (approximately 3 drops) of the lysed sample is added to the sample port in the assay cassette, and the 15-min assay incubation period is started. After the sample is added to the sample port, the fluid is absorbed by the sample pad. Liquid then flows by capillary action from the sample pad to a conjugate pad that contains additional reagents to condition the specimen and prepare it for optimal reactivity. While migrating through the conjugate pad, the diluted sample encounters detecting antibodies conjugated to the fluorescently labeled (europium) beads. The luminescent europium conjugate binds to any SARS/SARS-CoV-2 nucleocapsid antigen present in the sample and will ultimately produce a positive test result. The liquid moves along the test strip onto the nitrocellulose membrane containing two immobilized reagent lines [in order of sample contact: Test Line (anti-SARS-CoV-2 nucleocapsid antibody) and Control Line (anti-immunoglobulin antibodies)]. The sample mixture, including the detection antibody conjugate, will continue to migrate along the nitrocellulose membrane. The detection antibody conjugate will bind to the control line, forming a fluorescent line, to indicate the test was run correctly and establishes assay validity (if no control line is readable, the Halo reader will indicate an invalid test and prompt the user to repeat the test). The liquid will continue to be drawn toward the absorbent pad, which is placed after the nitrocellulose membrane, until the fluid has been exhausted from the sample and conjugate pads located at the beginning of the assay.

If SARS-CoV-2 nucleocapsid antigen is present in the sample, the europium reporter detecting antibody/antigen complex is captured by the test line antibodies, providing the Halo reader with a quantifiable signal that is subsequently analyzed by proprietary algorithms on the device. The intensities of the test and control lines are recorded relative to the overall range of the detector. The maximum value is set to 1 and the test and control line intensities are reported as a fraction of that value (e.g., a test line intensity of 0.1 would correspond to an intensity peak that was 10% of the full scale). Test line signal intensity above a pre-determined threshold and the presence of a control line indicates a SARS-CoV-2 antigen positive test result.

### Live Virus Testing

The average retention volume of the swabs was determined gravimetrically using simulated nasal matrix (SNM). A sample volume of 12 μl was determined to be the average amount of material retained by the ClearTip swabs (Yukon Medical, Durham, NC) used in the studies. Briefly, ten ClearTip swabs were unpackaged, individually weighed and added to separate tubes containing 0.5 mL of simulated nasal matrix^4^. The swabs were allowed to soak for 30–45 s and then removed from the liquid allowing any excess solution to drip off. The weight of the individual swabs including any residual SNM were recorded, and this information was used to determine the retained weight of the SNM captured on the swab. The values were averaged, converted to volumes, and multiplied by 0.5 to obtain the sample volume used for the analytical sensitivity (limit of detection) studies.

Previously prepared and frozen SNM was used for the study and was thawed and brought to room temperature before use. Similarly, SARS-CoV-2 stocks were thawed prior to use but were kept on ice during the procedure to preserve the sample integrity. These stocks were diluted with SNM to the appropriate concentration for a given experiment, and then 12 μl of virus-laden SNM was pipetted onto a ClearTip swab contained in a 1.5 mL tube. Separate tubes were prepared for performing replicates at each concentration. 300 μl of lysis buffer was then added to the tube, and the swab mixed by twirling and swirling in the lysis buffer for 15 s while pressing the swab tip against the sides of the tube. The swab was left in the lysis buffer for 1 min after mixing to lyse the viral particles, if any, in the sample. The test cassette was placed on a level surface and 80 μl of the solution was then added dropwise to the assay cassette via pipette. The assay was left to run for 15 min at controlled room temperature (15°C - 25°C). Following the 15-min incubation, the tests were read by inserting the cassette into the Halo reader and using a phone to obtain the test line intensities. In cases where a second reader was used, the assay was read within 2 min after the initial analysis. The preliminary limit of detection (LoD) was determined by the lowest concentration of SARS-CoV-2 at which all five test replicates were positive. Sensitivity was also measured for the delta variant by conducting 20 additional tests at the preliminary LoD concentration.

### Positive/Negative Threshold Determination

The positive/negative threshold was determined by analyzing a series of 10 samples of SNM with no SARS-CoV-2 present (Supplementary Table S1). The average and standard deviation of these samples were calculated, and the threshold was set at a value corresponding to the mean plus three times the standard deviation.

### Halo Reader Operation for Use in Laboratory Testing

The reader was removed from any packaging, placed on a level surface, and powered on by plugging in the supplied power cord. After a short boot-up sequence, a status light indicated that the reader was ready to accept a Bluetooth low energy connection. An iPhone 11 was provided with the HaloHost app preinstalled, which is a custom application for communication with the reader. After launching the app on the phone and connecting to the reader via Bluetooth, a sample identification number could be entered into the app to identify the test. Subsequent screens contained timers for the 1-min lysis buffer immersion and the 15-min assay incubation time. After the 15-min incubation time, the sample analysis could be initialized and once the data collection and analysis were complete, the test and control line intensities were displayed^5^ on the phone screen to be recorded. Two versions of the Halo reader were tested simultaneously. The first (Gen 1) was a functional prototype and the second (Gen 2) was a designed-for-manufacture (DFM) version.

### RT-PCR Testing of SARS-CoV-2 Variant Spiked SNM Samples at C2Sense LoD Concentration

To estimate RT-PCR Ct values of samples at the previously determined LoDs of the C2Sense Halo SARS-CoV-2 test, mock nasal samples at 1 ×, 0.1 ×, and 0.01 × the C2Sense LoD concentration were prepared for the SARS-CoV-2 isolates listed in Table 1. Ten-fold dilutions of virus stock in SNM were prepared for each isolate and 50 μl of sample added to the head of a dry nasal swab (unspiked SNM served as a negative control). To simulate a nasal swab collected for RT-PCR testing, the swab was transferred to a tube containing 3 ml of viral transport media (VTM), mixed by swirling, and left in the tube. A single nucleic acid extract was prepared from each VTM sample using the Qiagen QIAamp Viral RNA Mini Kit per the manufacturer's instructions, with a final elution using 140 μl of AVE buffer.

**Table 1:** Preliminary LoD in SNM—Halo Reader Live Virus in SNM.

**Isolate**	**Common variant name**	**Lineage**	**Halo preliminary LoD:**		**Halo preliminary LoD:**
			**Virus concentration in SNM**		**TCID_**50**_ per test**
			**TCID_**50**_/mL**	**Copies/ml (PCR determined)**	
USA-WA1/2020 (working stock 2)	Wild type	A	1.33 × 10^3^	1.4 × 10^6^	4.1
USA-WA1/2020 (working stock 6)	Wild type	A	3.16 × 10^2^	1.1 × 10^6^	1.0
USA/CA_CDC_5574/2020	Alpha	B.1.1.7	2.8 × 10^2^	2.3 × 10^6^	0.9
hCoV-19/South Africa/KRISP-K005325/2020	Beta	B.1.351	1.25 × 10^2^	1.0 × 10^6^	0.4
hCoV-19/Japan/TY7-503/2021	Gamma	P.1	8.94 × 10^2^	NA	2.7
hCoV-19/USA/PHC658/2021	Delta	B.1.617.2	3.08 × 10^2^	8.75 × 10^5^	1.0

*NA, not available*.

Nucleic acid extracts were tested on the CDC 2019-Novel Coronavirus (2019-nCoV) Real-Time RT-PCR Diagnostic Panel per the Instructions for Use (Rev 7, 07/21/2021). Each nucleic acid extract was tested in triplicate wells with the N1 primer/probe set, while a single well was used for the RNaseP endogenous control primer/probe set.

## Results and Discussion

### Setting the Positive/Negative Threshold

To establish the positive/negative cutoff value for the LoD studies, a total of ten (10) negative SNM replicates were tested and read after 15 mins on both the Gen 1 and Gen 2 instruments (1/100 dilution of SARS-CoV-2 in SNM was tested as a positive control). The results from the SNM testing are summarized in Supplementary Table S1. The Halo reader returns values from 0 to 1 depending on the intensity of the light emitted from the test and control lines. Using the criteria of the mean plus three standard deviations, the positive/negative cutoff values calculated for the Gen 1 (prototype) and Gen 2 (designed for manufacture) readers were 0.0328 and 0.0323, respectively. These cutoff values are specific to SNM for use in the subsequent LoD studies and may not be applicable to actual clinical samples.

### Determination of the Limit of Detection of C2Sense SARS-CoV-2 Assay

**Figure 2:**
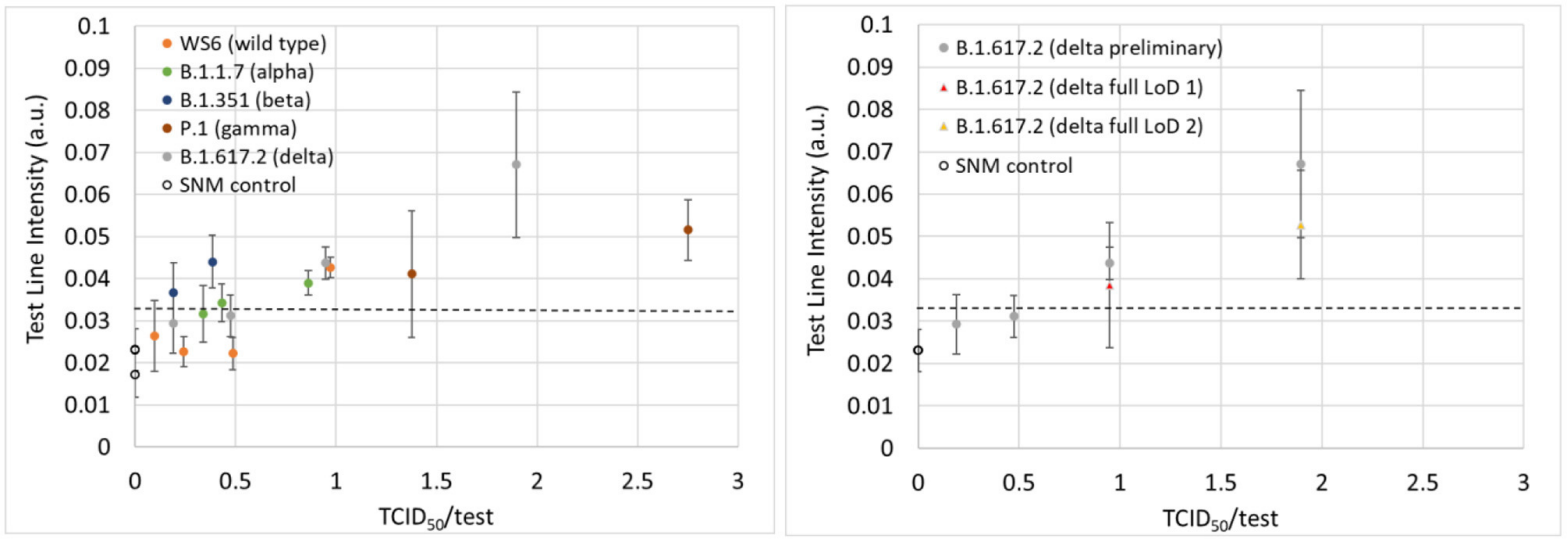
**(Left)** Test line intensities plotted as a function of the TCID_50_/test for the wild type and four variants from the preliminary LoD studies. **(Right)** The test line intensities for the delta variant for both the preliminary (gray data points) and confirmatory (yellow and red data points) LoD experiments. The dashed line represents the positive/negative threshold.

Preliminary LoD studies were performed using wild type and four variants, in addition to confirmatory determination of the LoD with delta variant live viral samples. The LoDs in units of TCID_50_/test are presented in Table 1 and ranged anywhere from 0.4 to 4.1 depending on the variant (experimental data can be found in Supplementary Tables S2–S20). The TCID_50_/test value is indicative of the amount of virus that is added to a test cassette for a given experiment. The values were calculated based on the concentration of the virus in SNM added to the swab (TCID_50_/mL), the volume of SNM added to the swab (12 μl), the dilution of that material by the addition of the lysis buffer (300 μl), and volume of diluted sample loaded onto the test cassette (80 μl). Data for the preliminary LoD studies displayed consistent performance across variants [see Figure 2 (left)] as well as consistent increases in signal when the amount of virus delivered to the assay was increased [Figure 2 (right)], which indicates that the test may have utility in providing a semi-quantitative output, similar to the cycle threshold in PCR testing. Additional tests were performed with recombinant protein to assess the ability of the test as a semi-quantitative measure of the amount of virus in each sample and the results are plotted in Supplementary Figure S2. Good linearity was observed in the 0–16 pg/ml regime as well as good operator-to-operator variability.

After preliminary LoD values were found, an experiment was performed to confirm the LoD for the delta variant. These experiments entailed running an additional 20 samples at the preliminary LoD concentration as well as five additional SNM samples with no virus present. The data from the initial LoD confirmation experiment at a TCID_50_/test value of 1 is contained in the supplementary information section (Supplementary Table S10). At this concentration, both the Gen 1 and Gen 2 readers incorrectly identified two (Gen 1) and four (Gen 2) of the 20 samples as negative, so the test was repeated at a higher concentration (two times the concentration of the preliminary LoD). At a fully diluted value of 1.9 TCID_50_/test (Supplementary Table S11), both readers were able to correctly identify 19/20 positive samples, thus meeting the FDA LoD criteria of ≥95% positivity rate at the limit of detection. After re-analyzing the sample incorrectly identified as negative, the Gen 2 reader successfully identified this sample as a positive sample, and correctly identified all samples thus achieving a sensitivity of 100% at the limit of detection. Preliminary limits of detection expressed in units of TCID_50_/mL, a measure of the concentration of virus in a sample, are reported for both the concentration contained in the 12 μl of virus containing SNM on the swab (Table 1, column 4) as well as on the amount of virus delivered to the assay (TCID_50_/test). The TCID_50_/test metric accounts for the dilution of the sample in 300 μl of lysis buffer as well as the amount of fluid added to the test cassette (80 μl). The TCID_50_/test values for virus types ranged from 0.4 for the beta variant to 4.1 for the wild type (WS2).

Given the extreme sensitivity of the Halo system, a comparative analysis was performed to assess the performance of the system vs. other commercially available assays. While the names of the manufacturers used in the comparison studies have been masked, at the time of submission of this manuscript, five (5) of the comparator LFAs evaluated (all except “E”) had received emergency use authorization (EUA) from the FDA. Two (2) of the comparator assays (C and D) use a fluorescent signal and reader system, similar to the C2Sense assay, while the other four (4) assays are traditional visually interpreted LFAs.

Table 2 ranks the performance of the seven (7) LFAs based on LoD for the different SARS-CoV-2 variants, with “1” being the most sensitive (Supplementary Table S21 summarizes the LoD results reported in TCID_50_/ml). The C2Sense Halo SARS-CoV-2 Test was the overall top performing assay, exhibiting the lowest LoD for all variants tested, with the exception of one of the kit lots from manufacturer “B” for the Lineage A isolate. Compared to the other assays, C2Sense Halo SARS-CoV2 test was 2X−39X more sensitive for detecting the B.1.1.7 variant; 3X−100X more sensitive for the B.1.351 variant; 5X−132X more sensitive for the P.1 variant; 3X−50X more sensitive for the B.1.617.2 variant; and 20X−80X more sensitive for the B.1.1.529 variant. It should be noted that the LoD studies described here used *in vitro* cultured virus spiked into SNM, and that the performance differences observed may or may not be indicative of clinical performance with real patient samples. Additionally, the other LFAs evaluated in the comparison studies are optimized for use with clinical nasal swab samples. Any assay optimization required to maximize performance of the C2Sense assay for use with clinical samples (e.g., minimize false positive results) could impact analytical sensitivity.

**Table 2:** SARS-CoV-2 lateral flow immunoassay performance rankings.

**Assay**	**Assay performance ranking by variant LoD**							**Average** **ranking**
	**A**	**A**	**B.1.1.7**	**B.1.351**	**P.1**	**B.1.617.2**	**B.1.1.529**	
C2Sense Halo	2	1	1	1	1	1	1	1.1
SARS-CoV-2
test*
B	1^a^	2^b^	2^a^	2^a^	2^b^	2 (tie)^c^	2 (tie)^c^	1.9
A^d^	3 (tie)	3 (tie)	3	3 (tie)	3 (tie)	2 (tie)	2 (tie)	2.7
E	5	5	4	3 (tie)	3 (tie)	2 (tie)	2 (tie)	3.4
F	3 (tie)	3 (tie)	5 (tie)	6 (tie)	7	7	TNP	5.2
C*	6	6	5 (tie)	5	6	6	4	5.4
D*	7	7	5 (tie)	6 (tie)	3 (tie)	5	5	5.4

*1 = Best performance; 5 = worst performance*.

*TNP, test not performed*.

* *Fluorescent based reader test*.

a *LoD from B1 kit lot used for comparison*.

b *LoD from B2 kit lot used for comparison*.

c *LoD from B3 kit lot used for comparison*.

d *Colorimetric version of the Halo SARS-CoV-2 test*.

In addition to testing against other commercially available rapid tests, the Halo system was tested against the gold standard for analytical detection of SARS-CoV-2. Results from the RT-PCR testing are summarized in Supplementary Table S22, and components are listed in Supplementary Table S23. At the C2Sense 1X LoD, all test replicates were positive on the CDC RT-PCR assay, with Ct values ranging from 30.9 (Lineage A WS2) to 34.4 (B.1.351). Average Ct value for all of the 1 × LoD samples was 32.4. The CDC RT-PCR assay detected all replicates of the 0.1 × LoD samples from four of the six (4/6) isolates [Lineage A (WS2), B.1.1.7, P.1, and B.1.617.2]. All 0.1 × LoD replicates were not detected for the Lineage A (WS6) and B.1.351 isolates. None of the 0.01 × LoD samples were positive on all replicates for any of the isolates. Although additional testing would be required to determine if similar performance differences would be observed with clinical patient specimens, these results suggest that the analytical sensitivity of the CDC 2019-Novel Coronavirus (2019-nCoV) Real-Time RT-PCR Diagnostic Panel is only approximately ten-fold more sensitive than the C2Sense Halo SARS-CoV-2 assay for detecting SARS-CoV-2 in mock nasal swab samples.

### Testing the Durability of the Halo Reader

Although not a comprehensive picture due to the limited number of readers tested, the similarity in threshold and results between the prototype (Gen 1) and designed-for-manufacture Halo reader (Gen 2) are extremely encouraging and point to minimal unit-to-unit variability. Additional tests will be performed on units to ensure this uniformity can be translated into mass produced units; however, it is shown here that the prototype and designed-for-manufacture reader perform so similarly. Another test of the quality of the unit was performed by acquiring data on a custom-made assay strip with europium-doped yttrium oxide to mimic the light emission from the europium-labeled beads in the real assay. Unlike the europium-labeled beads, the europium doped yttrium oxide does not readily photo-bleach when repeatedly exposed to the UV light used in the device. The performance of the system was found to be extremely stable even after an initial 100 exposures. A subsequent dataset was gathered the next day and an additional 1000 exposures were compiled. There was complete overlap between the two datasets (see Supplementary Figure S1) indicating that the system is also stable day-to-day. This indicates that the system is durable even though it was built from off-the-shelf parts and designed to be semi-disposable due to its low cost.

## Conclusions

Promising results were obtained from preliminary LoD studies performed with two different preparations of wild type SARS-CoV-2 (WS2 and WS6) and four different virus variants. In summary, preliminary limits of detection for wild type and four different variants were found to be between 0.4 and 4.1 TCID_50_/test. For the delta variant, which accounted for 95% of the cases worldwide^6^ when these experimental studies were being conducted, the preliminary limit of detection was found to be 1 TCID_50_/test and was confirmed at a level of 1.9 TCID_50_/test. The similarity of the preliminary limits of detection across the different variants suggests that the assay may be robust against a number of mutations.

Using the delta variant, 20 out of 20 samples were correctly identified (100% sensitivity) on the designed-for-manufacture reader (Gen 2) and a final LoD of 1.9 TCID_50_/test was obtained. These results highlight the sensitivity of the Halo platform. This low-cost system (cost of goods < $100 USD), comprised of readily available components for ease of scaling, can also facilitate reporting due to its cloud connectivity and non-human readable results. With the evolution of different virus strains, testing will undoubtedly be necessary for the foreseeable future. Tools such as the Halo platform, which can provide accurate results in a timely manner, can be a useful tool in providing critical information in a variety of settings including homes, schools, or clinics. In addition to standard fluorescent samples, Halo readers can also be adjusted to perform sophisticated delayed luminescence measurements as well as read colorimetric assays, all with an extremely low total cost. By simply swapping the label used for the diagnostic, any assay from any provider can in principle be rendered compatible with the system. Halo can also be modified for potential multiplex application using different fluorescent reporters and multiple test lines.

Antigen tests generally do not match the sensitivity reported by PCR tests. This is in part because antigen tests lack the target amplification that occurs during PCR. Theoretically, in a PCR test, after 35 cycles of amplification, the target can be amplified almost a billion-fold. In order to enhance the sensitivity of immunoassays, other methods have been employed, i.e., signal amplification (MSD platform) or more sensitive reporters (fluorescence). It has been shown that PCR-level sensitivity can be achieved by an antigen test using signal amplification (12). The drawback in using fluorescence reporters is that it cannot be visualized by the human eye and therefore requires a reader with a light source for excitation and emission of fluorescence that can be measured. Here we describe a very low-cost reader and assay that achieves considerable sensitivity, which can be easily modified to read other types of lateral flow assays (e.g., colorimetric or fluorescence lifetime-based). The performance of the Halo system was tested against six different commercially available assays and was found to be the most sensitive for all virus strains except for the wild-type strain, where it was the most sensitive test compared to one kit lot from manufacturer “B,” but second most sensitive when compared to a second kit lot from manufacturer “B.” In addition, the Halo reader can be configured to provide semi-quantitative results, akin to PCR Ct values, which could be indicative of viral load differences between patients. Moreover, data from an electronically read, sensitive lateral flow assay (i.e., Halo) can be combined with PCR assay data to make reasonable calls on infectivity and make more informed public health decisions early in the infection cycle. Similarly, sensitive assays such as the one described here can be a good test for use in monitoring individuals during the infectious period. Our future efforts will focus on these aspects of making Halo a universal reader.

## Data Availability Statement

The original contributions presented in the study are included in the article/Supplementary Material, further inquiries can be directed to the corresponding author/s.

## Author Contributions

JW, JC, and RD designed and developed the Halo Reader. Maxim Biomedical (JMa, JMaa, KP, and NO) designed and produced the COVID assay. MRI Global (JS and JB) tested the performance of the system. DoD (BG and SS) provided funding and technical support for the work. All authors participated in writing the manuscript. All authors contributed to the article and approved the submitted version.

## Funding

This work was supported by Department of Defense Contract (FA807518D0017) through a Subcontract (863-111084-19) with MRI Global.

## Conflict of Interest

SS and BG were employed by the DoD. JB, JS, NH, and MM were employed by MRIGlobal. This study received funding from the DoD through an MRIGlobal subcontract. The DoD did not have any involvement in the study design. MRIGlobal and C2Sense designed the study protocol. C2Sense designs and sells diagnostic tools. Maxim Biological develops and sells lateral flow assays. The remaining authors declare that the research was conducted in the absence of any commercial or financial relationships that could be construed as a potential conflict of interest.

## Publisher's Note

All claims expressed in this article are solely those of the authors and do not necessarily represent those of their affiliated organizations, or those of the publisher, the editors and the reviewers. Any product that may be evaluated in this article, or claim that may be made by its manufacturer, is not guaranteed or endorsed by the publisher.

## Footnotes

^1^https://www.who.int/director-general/speeches/detail/who-director-general-s-opening-remarks-at-the-media-briefing-on-covid-19-−11-march-2020 (accessed April 1, 2022).

^2^https://www.who.int/emergencies/diseases/novel-coronavirus-2019 (accessed July 12, 2021).

^3^https://covid19.who.int/ (accessed July 12, 2021).

^4^Simulated nasal matrix: phosphate buffer solution containing glycerol (15%), porcine mucin (2.5%), and human blood (1%).

^5^When used as a diagnostic tool, “positive,” “negative,” or “invalid” would be displayed instead of test and control line intensities.

^6^https://nextstrain.org/ncov/gisaid/global (accessed November 30, 2021).
