# Supplementary material for: Halo—A Universal Fluorescence Reader Based Threat Agent Detection Platform—A Proof of Concept Study Using SARS-CoV-2 Assays
Source: Front Public Health. 2022 Apr 12;10:852083. doi: 10.3389/fpubh.2022.852083 (PMC9039038; doi:10.3389/fpubh.2022.852083)
Supplement: Supplementary file 1 [file Data_Sheet_1.pdf]

1 Supplementary Material

Table S1. Test Line Intensities – Negative SNM Cutoff Testing

Sample	Replicate	Reader	
		Gen 1	Gen 2
SNM (unspiked)	1	0.0221	0.0176
	2	0.0224	0.0221
	3	0.0177	0.0193
	4	0.015	0.0169
	5	0.0263	0.0248
	6	0.0239	0.0246
	7	0.0259	0.0245
	8	0.0163	0.0148
	9	0.0226	0.0116
	10	0.0162	0.0166
1/100 SARS-CoV-2 (WS6)	N/A	0.4259	0.4057

N/A = Not Applicable.
Highlighted cells were called "Positive" based on cutoffs of 0.0328 and 0.0323 for the Gen 1 and Gen 2 readers, respectively.

Table S2. Preliminary LoD Determination in SNM – (wild type virus)

Sample	$TCID_{50}$/mL (in SNM)	Copies/mL (in SNM)	Replicates Tested	# Positive Calls
SNM (unspiked)	N/A	N/A	5	0
1/1000 SARS-CoV-2 (WS2)	5.31×10^3	5.4×10^6	5	5
1/10,000 SARS-CoV-2 (WS2)	5.31×10^2	5.4×10^5	5	0
1/2000 SARS-CoV-2 (WS2)	2.66×10^3	2.7×10^6	TNP	TNP
1/4000 SARS-CoV-2 (WS2)	1.33×10^3	1.4×10^6	5	5
1/8000 SARS-CoV-2 (WS2)	6.64×10^2	6.8×10^5	5	2

N/A = Not Applicable.
TNP = Test Not Performed.
Gen 1 reader used.

Table S3. Test Line Intensities - Preliminary LoD Determination in SNM

Sample	Replicate 1	Replicate 2	Replicate 3	Replicate 4	Replicate 5	Average	SD
SNM (unspiked)	0.0036	0.0114	0.0088	0.0036	0.0066	0.0068	0.003379
1/1000 SARS-CoV-2 (WS2)	0.2074	0.1715	0.1870	0.1736	0.1616	0.1802	0.017687
1/10,000 SARS-CoV-2 (WS2)	0.0221	0.0239	0.0166	0.0264	0.0239	0.0226	0.003677
1/4000 SARS-CoV-2 (WS2)	0.0439	0.0494	0.0453	0.0441	0.0495	0.0464	0.0028
1/8000 SARS-CoV-2 (WS2)	0.0299	0.0170	0.0354	0.0251	0.0333	0.0281	0.007345

Highlighted cells were called "Positive" by the Halo instrument algorithm.
Gen 1 reader used.

Table S4. Preliminary LoD Determination in SNM – Gen 1 and Gen 2 readers

Sample	TCID50/mL (in SNM)	Copies/mL (in SNM)	Replicates Tested	# Positive Calls	
				Gen 1 Reader	Gen 2 Reader
SNM (unspiked)	N/A	N/A	5	1[a]	0
1/10,000 SARS-CoV-2 (WS6)	3.16×10^{2}	1.1×10^{6}	5	5	5
1/100,000 SARS-CoV-2 (WS6)	3.16×10^{1}	1.1×10^{5}	5	1	1
1/20,000 SARS-CoV-2 (WS6)	1.58×10^{2}	5.5×10^{5}	5	0	0
1/40,000 SARS-CoV-2 (WS6)	7.90×10^{1}	2.8×10^{5}	5	1	0

N/A = Not Applicable.

[a] Negative on second read.

Results called “Positive” based on cutoffs of 0.0328 and 0.0323 for the Gen 1 and Gen 2 readers, respectively.

Table S5. Test Line Intensities - Preliminary LoD Determination in SNM (Gen 1 Reader)

Sample	Replicate 1	Replicate 2	Replicate 3	Replicate 4	Replicate 5	Average	SD
SNM (unspiked)	0.0224	0.0187	0.0207	0.0073	0.1184[a]	0.0375	0.00682
1/10,000 SARS-CoV-2 (WS6)	0.0396	0.0477	0.0442	0.0449	0.0417	0.0436	0.003101
1/100,000 SARS-CoV-2 (WS6)	0.0257	0.0277	0.0516	0.0266	0.0237	0.0311	0.011576
1/20,000 SARS-CoV-2 (WS6)	0.0186	0.0173	0.0288	0.0245	0.0297	0.0238	0.005692
1/40,000 SARS-CoV-2 (WS6)	0.0217	0.0302	0.0216	0.0284	0.0328	0.0269	0.005076

N/A = Not Applicable.

Highlighted cells were called “Positive” based on a cutoff of 0.0328 for the Gen 1 reader.

[a] Negative (0.0266) on second read.

Table S6. Test Line Inensities - Preliminary LoD Determination in SNM (Gen 2 Reader)

Sample	Replicate 1	Replicate 2	Replicate 3	Replicate 4	Replicate 5	Average	SD
SNM (unspiked)	0.0218	0.0169	0.0226	0.0093	0.0152	0.0172	0.005404
1/10,000 SARS-CoV-2 (WS6)	0.0404	0.0425	0.0466	0.0427	0.0413	0.0427	0.002372
1/100,000 SARS-CoV-2 (WS6)	0.0213	0.0245	0.0412	0.0247	0.0207	0.0265	0.008426
1/20,000 SARS-CoV-2 (WS6)	0.0197	0.0169	0.0262	0.0238	0.0244	0.0222	0.003799
1/40,000 SARS-CoV-2 (WS6)	0.0225	0.0262	0.0232	0.0170	0.0244	0.0227	0.003461

N/A = Not Applicable.

Highlighted cells were called “Positive” based on a cutoff of 0.0323 for the Gen 2 reader.

Table S7. B.1.617.2 Isolate Preliminary LoD Determination in SNM

Sample	TCID50/mL (in SNM)	Copies/mL (in SNM)	Replicates Tested	# Positive Calls Gen 1 Reader	# Positive Calls Gen 2 Reader
SNM (unspiked)	N/A	N/A	5	0	0
1/1000 SARS-CoV-2	6.16×10^3	1.75×10^7	5	5	5
1/10,000 SARS-CoV-2	6.16×10^2	1.75×10^6	5	5	5
1/100,000 SARS-CoV-2	6.16×10^1	1.75×10^5	5	1	1
1/20,000 SARS-CoV-2	3.08×10^2	8.75×10^5	5	5	5
1/40,000 SARS-CoV-2	1.54×10^2	4.38×10^5	5	2	3

N/A = Not Applicable.
Results called “Positive” based on cutoffs of 0.0328 and 0.0323 for the Gen 1 and Gen 2 readers, respectively.

Table S8. Test Line Intensities - B.1.617.2 Isolate Preliminary LoD (Gen 1 Reader)

Sample	Replicate 1	Replicate 2	Replicate 3	Replicate 4	Replicate 5	Average	SD
SNM (unspiked)	0.0196	0.0219	0.0176	0.0219	0.0276	0.0217	0.003745
1/1000 SARS-CoV-2	0.3314	0.3287	0.3805	0.3159	0.3473	0.3408	0.024865
1/10,000 SARS-CoV-2	0.2115	0.0732	0.0590	0.0587	0.0931	0.0991	0.064383
1/100,000 SARS-CoV-2	0.0184	0.0255	0.0282	0.0214	0.0437	0.0274	0.009835
1/20,000 SARS-CoV-2	0.0417	0.0453	0.0424	0.1215	0.0477	0.0597	0.034619
1/40,000 SARS-CoV-2	0.0287	0.0286	0.0285	0.0501	0.0400	0.0352	0.009692

N/A = Not Applicable.

Highlighted cells were called “Positive” based on a cutoff of 0.0328 for the Gen 1 reader.

Table S9. Test Line Intensities - B.1.617.2 Isolate Preliminary LoD (Gen 2 Reader)

Sample	Replicate 1	Replicate 2	Replicate 3	Replicate 4	Replicate 5	Average	SD
SNM (unspiked)	0.0190	0.0229	0.0175	0.0276	0.0284	0.0231	0.004913
1/1000 SARS-CoV-2	0.3139	0.3052	0.3491	0.3117	0.3152	0.3190	0.01725
1/10,000 SARS-CoV-2	0.1992	0.0715	0.0538	0.0533	0.0898	0.0935	0.060956
1/100,000 SARS-CoV-2	0.0223	0.0275	0.0289	0.0268	0.0410	0.0293	0.006992
1/20,000 SARS-CoV-2	0.0424	0.0445	0.0394	0.1156	0.0485	0.0581	0.032325
1/40,000 SARS-CoV-2	0.0325	0.0329	0.0312	0.0230	0.0363	0.0312	0.004945

N/A = Not Applicable.

Highlighted cells were called “Positive” based on a cutoff of 0.0323 for the Gen 2 reader.

Table S10. LoD Confirmation (1/20,000 dilution) – B.1.617.2 Isolate (Gen 1 and Gen 2 Readers)

Sample	$TCID_{50}$/mL (in SNM on the swab)	Copies/mL (in SNM on the swab)	Test Line Intensity	
			Gen 1 Reader	Gen 2 Reader
SNM (unspiked)	N/A	N/A	0.0258	*
SNM (unspiked)	N/A	N/A	0.0247	*
SNM (unspiked)	N/A	N/A	0.0237	0.0241
SNM (unspiked)	N/A	N/A	0.0203	0.0219
SNM (unspiked)	N/A	N/A	0.0204	0.0132
1/20,000 SARS-CoV-2	3.08×10^2	8.75×10^5	0.2076	0.1987
1/20,000 SARS-CoV-2	3.08×10^2	8.75×10^5	0.0475	0.0461
1/20,000 SARS-CoV-2	3.08×10^2	8.75×10^5	0.0387	0.0399
1/20,000 SARS-CoV-2	3.08×10^2	8.75×10^5	0.0470	0.0371
1/20,000 SARS-CoV-2	3.08×10^2	8.75×10^5	0.0410	0.0390
1/20,000 SARS-CoV-2	3.08×10^2	8.75×10^5	0.0365	0.0222
1/20,000 SARS-CoV-2	3.08×10^2	8.75×10^5	0.1492	0.1460
1/20,000 SARS-CoV-2	3.08×10^2	8.75×10^5	0.0458	0.0429
1/20,000 SARS-CoV-2	3.08×10^2	8.75×10^5	0.0343	0.0329
1/20,000 SARS-CoV-2	3.08×10^2	8.75×10^5	0.1238	0.1288
1/20,000 SARS-CoV-2	3.08×10^2	8.75×10^5	0.0430	0.0444
1/20,000 SARS-CoV-2	3.08×10^2	8.75×10^5	0.0355	0.0291
1/20,000 SARS-CoV-2	3.08×10^2	8.75×10^5	0.0348	0.0354
1/20,000 SARS-CoV-2	3.08×10^2	8.75×10^5	0.0400	0.0390
1/20,000 SARS-CoV-2	3.08×10^2	8.75×10^5	0.0391	0.0403
1/20,000 SARS-CoV-2	3.08×10^2	8.75×10^5	0.0276	0.0256
1/20,000 SARS-CoV-2	3.08×10^2	8.75×10^5	0.0269	0.0246
1/20,000 SARS-CoV-2	3.08×10^2	8.75×10^5	0.0335	0.0359
1/20,000 SARS-CoV-2	3.08×10^2	8.75×10^5	0.0353	0.0892
1/20,000 SARS-CoV-2	3.08×10^2	8.75×10^5	0.0336	0.0323

N/A = Not Applicable.
Highlighted cells were called “Positive” based on a cutoffs of 0.0328 and 0.0323 for the Gen 1 and Gen 2 readers, respectively.
*Test line intensity not recorded on worksheet (control line intensity recorded in error) - assumed negative based on Gen 1 reader result.

Table S11. LoD Confirmation (1/10,000 dilution) – B.1.617.2 Isolate (Gen 1 and Gen 2 Readers)

Sample	$TCID_{50}$/mL (in SNM on the swab)	Copies/mL (in SNM on the swab)	Test Line Intensity	
			Gen 1 Reader	Gen 2 Reader
SNM (unspiked)	N/A	N/A	0.0276	0.0223
SNM (unspiked)	N/A	N/A	0.0191	0.0190
SNM (unspiked)	N/A	N/A	0.0196	0.0220
SNM (unspiked)	N/A	N/A	0.0146	0.0156
SNM (unspiked)	N/A	N/A	0.0119	0.0533[a]
1/10,000 SARS-CoV-2	6.16×10^2	1.75×10^6	0.0589	0.0546
1/10,000 SARS-CoV-2	6.16×10^2	1.75×10^6	0.0401	0.0343
1/10,000 SARS-CoV-2	6.16×10^2	1.75×10^6	0.0380	0.0387
1/10,000 SARS-CoV-2	6.16×10^2	1.75×10^6	0.0396	0.0364
1/10,000 SARS-CoV-2	6.16×10^2	1.75×10^6	0.0533	0.0481
1/10,000 SARS-CoV-2	6.16×10^2	1.75×10^6	0.0498	0.0500
1/10,000 SARS-CoV-2	6.16×10^2	1.75×10^6	0.0535	0.0518
1/10,000 SARS-CoV-2	6.16×10^2	1.75×10^6	0.0527	0.0547
1/10,000 SARS-CoV-2	6.16×10^2	1.75×10^6	0.0559	0.0549
1/10,000 SARS-CoV-2	6.16×10^2	1.75×10^6	0.0502	0.0515
1/10,000 SARS-CoV-2	6.16×10^2	1.75×10^6	0.0489	0.0456
1/10,000 SARS-CoV-2	6.16×10^2	1.75×10^6	0.0407	0.000[b]
1/10,000 SARS-CoV-2	6.16×10^2	1.75×10^6	0.0538	0.0520
1/10,000 SARS-CoV-2	6.16×10^2	1.75×10^6	0.0568	0.0612
1/10,000 SARS-CoV-2	6.16×10^2	1.75×10^6	0.0530	0.0502
1/10,000 SARS-CoV-2	6.16×10^2	1.75×10^6	0.0458	0.0437
1/10,000 SARS-CoV-2	6.16×10^2	1.75×10^6	0.0609	0.0641
1/10,000 SARS-CoV-2	6.16×10^2	1.75×10^6	0.0534	0.0516
1/10,000 SARS-CoV-2	6.16×10^2	1.75×10^6	0.0590	0.0567
1/10,000 SARS-CoV-2	6.16×10^2	1.75×10^6	I	0.0586

N/A = Not Applicable.
I = Invalid (failure to read control line).
Highlighted cells were called "Positive" based on cutoffs of 0.0328 and 0.0323 for the Gen 1 and Gen 2 readers, respectively.
[a] Negative when reread on both readers: 0.0131 and 0.0167 on Gen 1 and Gen 2, respectively.
[b] 0.0960 (positive) when read a second time.

Table S12. B.1.1.7 Isolate Preliminary LoD Determination in SNM

Sample	$TCID_{50}$/mL (in SNM)	Copies/mL (in SNM)	Replicates Tested	# Positive Calls	
				Gen 1 Reader	Gen 2 Reader
SNM (unspiked)	N/A	N/A	TNP	TNP	TNP
1/100,000 SARS-CoV-2	1.1×10^2	9.0×10^5	5	1	1
1/40,000 SARS-CoV-2	2.8×10^2	2.3×10^6	5	5	5
1/80,000 SARS-CoV-2	1.4×10^2	1.1×10^6	5	3	3

N/A = Not Applicable.
TNP = Test Not Performed (testing performed same day as B.1.617.2 LoD confirmation).
Results called "Positive" based on cutoffs of 0.0328 and 0.0323 for the Gen 1 and Gen 2 readers, respectively.

Table S13.Test Line Intensities - B.1.1.7 Isolate Preliminary LoD (Gen 1 Reader)

Sample	Replicate 1	Replicate 2	Replicate 3	Replicate 4	Replicate 5	Average	SD
1/100,000 SARS-CoV-2	0.0323	0.0426	0.0287	0.0304	0.0269	0.0322	0.006159
1/40,000 SARS-CoV-2	0.0412	0.0379	0.0499	0.0432	0.0557	0.0456	0.007157
1/80,000 SARS-CoV-2	0.0294	0.0327	0.0332	0.0419	0.0373	0.0349	0.004815

N/A = Not Applicable.

Highlighted cells were called "Positive" based on a cutoff of 0.0328 for the Gen 1 reader.

Table S14.Test Line Intensities - B.1.1.7 Isolate Preliminary LoD (Gen 2 Reader)

Sample	Replicate 1	Replicate 2	Replicate 3	Replicate 4	Replicate 5	Average	SD
1/100,000 SARS-CoV-2	0.0304	0.0432	0.0280	0.0308	0.0257	0.0316	0.00679
1/40,000 SARS-CoV-2	0.0386	0.0377	0.0434	0.0397	0.0357	0.0390	0.002854
1/80,000 SARS-CoV-2	0.0284	0.0363	0.0309	0.0397	0.0357	0.0342	0.004512

N/A = Not Applicable.

Highlighted cells were called "Positive" based on a cutoff of 0.0323 for the Gen 2 reader.

Table S15. B.1.351 Isolate Preliminary LoD Determination in SNM

Sample	$TCID_{50}$/mL (in SNM)	Copies/mL (in SNM)	Replicates Tested	# Positive Calls	
				Gen 1 Reader	Gen 2 Reader
SNM (unspiked)	N/A	N/A	TNP	TNP	TNP
1/10,000 SARS-CoV-2	1.25×10^3	1.0×10^7	5	5	5
1/100,000 SARS-CoV-2	1.25×10^2	1.0×10^6	5	5	5
1/200,000 SARS-CoV-2	6.25×10^1	5.0×10^5	5	2	4

N/A = Not Applicable.

TNP = Test Not Performed (testing performed same day as B.1.617.2 LoD confirmation).

Results called "Positive" based on cutoffs of 0.0328 and 0.0323 for the Gen 1 and Gen 2 readers, respectively.

Table S16.Test Line Intensities - B.1.351 Isolate Preliminary LoD (Gen 1 Reader)

Sample	Replicate 1	Replicate 2	Replicate 3	Replicate 4	Replicate 5	Average	SD
1/10,000 SARS-CoV-2	0.1737	0.1790	0.3509	0.3496	0.2255	0.2557	0.088599
1/100,000 SARS-CoV-2	0.0594	0.0416	0.0378	0.0437	0.0473	0.0460	0.008263
1/200,000 SARS-CoV-2	0.0345	I	0.0322	0.0442	0.0281	0.0348	0.006833

N/A = Not Applicable.

I = Invalid (failure to read control strip). Negative (0.0187) on repeat read.

Highlighted cells were called "Positive" based on a cutoff of 0.0328 for the Gen 1 reader.

Table S17. Test Line Intensities - B.1.351 Isolate Preliminary LoD (Gen 2 Reader)

Sample	Replicate 1	Replicate 2	Replicate 3	Replicate 4	Replicate 5	Average	SD
1/10,000 SARS-CoV-2	0.1713	0.1665	0.3442	0.3095	0.2171	0.2417	0.081116
1/100,000 SARS-CoV-2	0.0532	0.0365	0.0404	0.0441	0.0458	0.0440	0.006267
1/200,000 SARS-CoV-2	0.0369	0.0327	0.0317	0.0490	0.0331	0.0367	0.007164

N/A = Not Applicable.

Highlighted cells were called “Positive” based on a cutoff of 0.0323 for the Gen 2 reader.

Table S18. P.1 Isolate Preliminary LoD Determination in SNM

Sample	$TCID_{50}$/mL (in SNM)	Copies/mL (in SNM)	Replicates Tested	# Positive Calls	
				Gen 1 Reader	Gen 2 Reader
SNM (unspiked)	N/A	N/A	TNP	TNP	TNP
1/100,000 SARS-CoV-2	8.94×10^2	NA	5	5	5
1/200,000 SARS-CoV-2	4.47×10^2	NA	5	4	4

N/A = Not Applicable.

NA = Not Available.

TNP = Test Not Performed (testing performed same day as B.1.617.2 LoD confirmation).

Results called “Positive” based on cutoffs of 0.0328 and 0.0323 for the Gen 1 and Gen 2 readers, respectively.

Table S19.Test Line Intensities – P.1 Isolate Preliminary LoD (Gen 1 Reader)

Sample	Replicate 1	Replicate 2	Replicate 3	Replicate 4	Replicate 5	Average	SD
1/100,000 SARS-CoV-2	0.0661	0.0420	0.0463	0.0512	0.0487	0.0509	0.00917
1/200,000 SARS-CoV-2	0.1032	0.0269	0.0440	0.0346	0.0369	0.0491	0.030841

N/A = Not Applicable.

Green highlighted cells were called “Positive” based on a cutoff of 0.0328 for the Gen 1 reader.

Table S20. Test Line Intensities - P.1 Isolate Preliminary LoD (Gen 2 Reader)

Sample	Replicate 1	Replicate 2	Replicate 3	Replicate 4	Replicate 5	Average	SD
1/100,000 SARS-CoV-2	0.0637	0.0491	0.0469	0.0524	0.0458	0.0637	0.007229
1/200,000 SARS-CoV-2	0.0664	0.0299	0.0430	0.0323	0.0338	0.0664	0.014999

N/A = Not Applicable.

Highlighted cells were called “Positive” based on a cutoff of 0.0323 for the Gen 2 reader.

Table S21 summarizes the LoD results reported in $TCID_{50}$/mL. For comparison, LoD results from six (6) other commercially available SARS-CoV-2 lateral flow immunoassays tested in an analogous fashion (virus stock diluted in simulated nasal matrix) are included. With the exception of the P.1 isolate, the same SARS-CoV-2 working stocks were used for LoD testing on all of the lateral flow immunoassays (a different P.1 stock was used for the C2Sense LoD studies).

Table S21.SARS-CoV-2 Lateral Flow Immunoassay Comparison – LoD by Variant ($TCID_{50}$/mL Titer).

Assay	SARS-CoV-2 Lineage - LoD ($TCID_{50}$/mL)					
	A	B.1.1.7	B.1.351	P.1	B.1.617.2	B.1.1.529
C2Sense Halo SARS-CoV-2 Test*	1.33×10^3	2.8×10^2	1.25×10^2	[a]8.94×10^2	6.16×10^2	2.59×10^0
A[1]	5.31×10^3	2.8×10^3	3.13×10^3	1.18×10^4	1.54×10^3	5.18×10^1
B1[b]	6.64×10^2	5.5×10^2	3.13×10^2	TNP	TNP	TNP
B2[b]	2.66×10^3	TNP	TNP	4.73×10^3	TNP	TNP
B3[b]	TNP	TNP	TNP	TNP	1.54×10^3	5.18×10^1
C*	2.66×10^4	1.1×10^4	6.25×10^3	4.73×10^4	1.54×10^4	1.04×10^2
D*	5.31×10^4	1.1×10^4	1.25×10^4	1.18×10^4	6.16×10^3	2.07×10^2
E	1.33×10^4	5.5×10^3	3.13×10^3	1.18×10^4	1.54×10^3	5.18×10^1
F	5.31×10^3	1.1×10^4	1.25×10^4	1.18×10^5	3.08×10^4	N/D[c]

TNP = Test Not Performed.
[1] Colorimetric version of the Halo SARS-CoV-2 test
* Fluorescence-based reader test.
[a] Different P.1 virus stock used for C2Sense LoD determination.
[b] Three different test kit lots from manufacturer B.
[c] Not Determined – all negative control replicates were positive, therefore an LoD could not be determined.

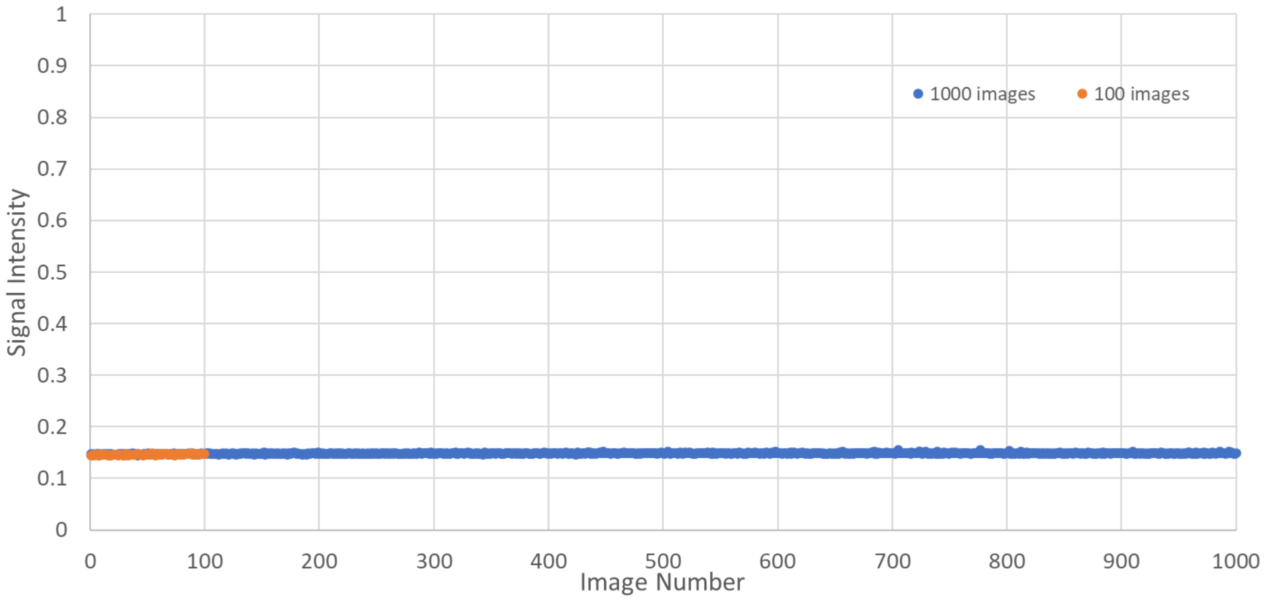

Figure S1: Halo reader durability testing. Using a non-photobleachable test strip, the reader was used to acquire a series of 100 and then 1000 images 16 hours later. The analysis was repeatable and unchanging over time indicating the system is robust and can be heavily used without any degradation in performance.

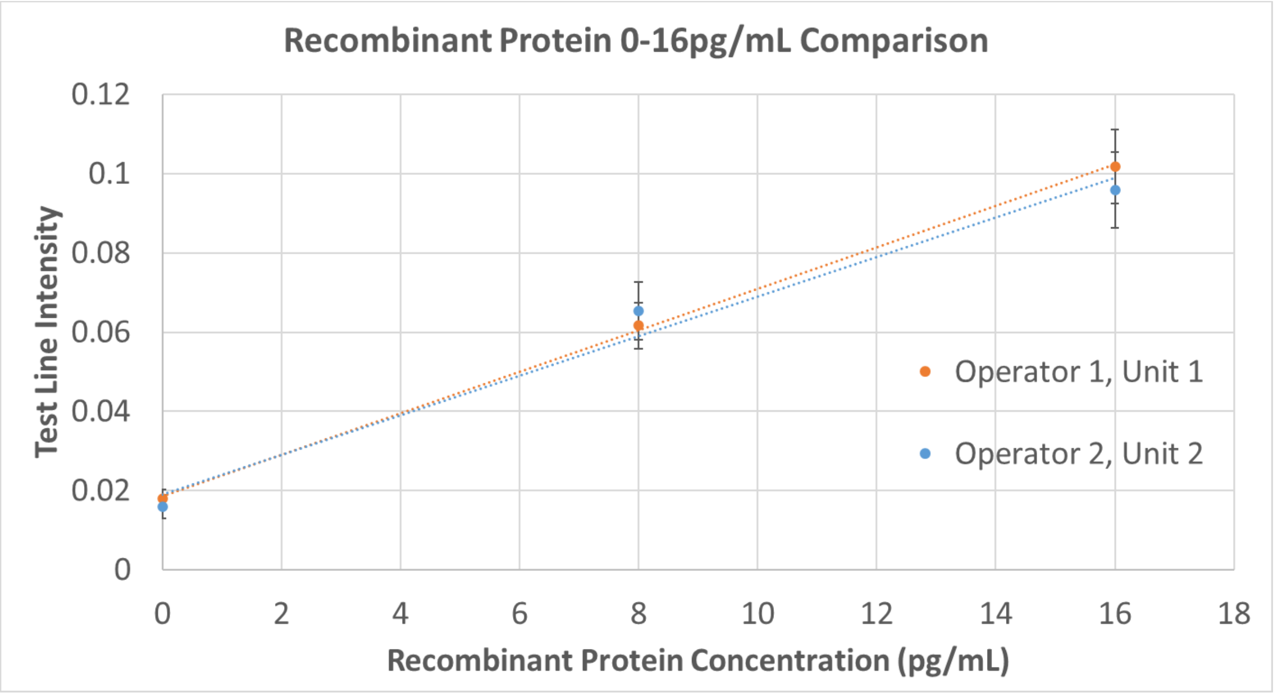

Figure S2: Linearity testing with recombinant protein. Recombinant protein samples were prepared using lysis buffer as a matrix. 5 samples at concentrations of 8 and 16 picogram per milliliter samples were prepared and run by two different operators on two different Halo readers on two different days. Lysis buffer with no recombinant protein were run as 0 pg/mL samples. The average and standard deviations plotted above for both datasets show good agreement and indicate highly linear behavior in this concentration regime.
